# The Pandemic Paranoia Scale (PPS): factor structure and measurement invariance across languages

**DOI:** 10.1017/S0033291721004633

**Published:** 2023-04

**Authors:** J. L. Kingston, B. Schlier, L. Ellett, S. H. So, B. A. Gaudiano, E. M. J. Morris, T. M. Lincoln

**Affiliations:** 1 Royal Holloway, University of London, London, UK; 2 Department of Clinical Psychology and Psychotherapy, University of Hamburg, Hamburg, Germany; 3 Department of Psychology, The Chinese University of Hong Kong, Shatin, Hong Kong; 4 Department of Psychiatry and Human Behavior, Brown University, Providence, RI, USA; 5 School of Psychology and Public Health, La Trobe University, Melbourne, Australia

**Keywords:** Conspiracy beliefs, COVID-19 pandemic, general population, international, paranoia

## Abstract

**Background:**

Globally, the corona virus disease 2019 (COVID-19) pandemic has created an interpersonally threatening context within which other people have become a source of possible threat. This study reports on the development and validation of a self-report measure of pandemic paranoia; that is, heightened levels of suspicion and mistrust towards others due to the COVID-19 pandemic.

**Methods:**

An international consortium developed an initial set of 28 items for the Pandemic Paranoia Scale (PPS), which were completed by participants from the UK (*n* = 512), USA (*n* = 535), Germany (*n* = 516), Hong Kong (*n* = 454) and Australia (*n* = 502) using stratified quota sampling (for age, sex and educational attainment) through Qualtrics and translated for Germany and Hong Kong.

**Results:**

Exploratory factor analysis in the UK sample suggested a 25-item, three-factor solution (persecutory threat; paranoid conspiracy and interpersonal mistrust). Confirmatory factor analysis (CFA) on the remaining combined sample showed sufficient model fit in this independent set of data. Measurement invariance analyses suggested configural and metric invariance, but no scalar invariance across cultures/languages. A second-order factor CFA on the whole sample indicated that the three factors showed large loadings on a common second-order pandemic paranoia factor. Analyses also supported the test–retest reliability and internal and convergent validity.

**Conclusion:**

The PPS offers an internationally validated and reliable method for assessing paranoia in the context of a pandemic. The PPS has the potential to enhance our understanding of the impact of the pandemic, the nature of paranoia and to assist in identifying and supporting people affected by pandemic-specific paranoia.

## Introduction

Paranoia, the belief that others will intentionally cause one harm (Freeman & Garety, 2000), is common in the general population with approximately 28% of individuals reporting the elevated levels of paranoid thinking in everyday life (Freeman et al., 2019). Paranoia can be conceptualised as existing on a continuum, ranging from mild concerns of suspicion and distrust of others, to less commonly reported delusions of persecution, which are more typical in clinical groups (Freeman et al., 2005). Deciphering the intentions of others and making judgements as to whether others can be trusted is a complex human process, which is influenced by individual and environmental factors, and their interaction. Stress-vulnerability models of paranoid thinking emphasise the key role of high stress environments (e.g. victimisation, minority status and social maladjustment), affective states (e.g. fear, anger, anxiety and depression) and cognitive mechanisms (e.g. self-representations, social cognition and mentalisation) in understanding the development of paranoid thinking (e.g. Freeman, Bentall, & Garety, 2008; Garety, Kuipers, Fowler, Freeman, & Bebbington, 2001; Preti & Cella, 2010; van Os, Kenis, & Rutten, 2010). According to Preti and Cella's (2010) human heuristic account, paranoia helps an individual manage uncertainty during stressful situations. Stress, triggered by environmental factors and affective states, is thought to enhance the probability that neutral stimuli are viewed as threatening, which functions to avoid possible harm whilst not missing possible social gain. Under these conditions, interpretations and decision making over-relies on paranoid-based interpretations of experience.

In January 2019, the corona virus disease 2019 (COVID-19) pandemic introduced a rapidly intensifying threat into the lives of people across the world, which has had a substantial effect on the physical and psychological wellbeing of many people (e.g. Chandola, Kumari, Booker, & Benzeval, 2020; Ran et al., 2020). The pandemic has created an environment that contains many of the characteristics that we know give rise to paranoia in the general population such as stress, worry, social detachment and isolation, loss of work, anxiety and depression (e.g. Lamster, Lincoln, Nittel, Rief, & Mehl, 2016; Olfson et al., 2002; Saarinen et al., 2020; Startup, Freeman, & Garety, 2007). Furthermore, and with reference to paranoia, it has introduced a heightened state of fear and anxiety as to whether others’ actions and intentions can be trusted. Studies examining paranoia during the pandemic have shown: that exposure to information about the pandemic is associated with paranoid thinking (Jaspal, Lopes, & Lopes, 2020), that paranoid thinking is higher than pre-established norms in Chinese students (Ruichen, 2020) and that young people, in particular, are likely to be experiencing paranoia in response to the pandemic (Lopes, Bortolon, & Jaspal, 2020). At the more severe end of the spectrum, Hu, Su, Qiao, Zhu, and Zhou (2020) reported a 25% increase in incident cases of psychosis compared to the previous year, which authors attributed to psychosocial stress and physical distancing.

Existing research on paranoia during the pandemic has utilised measures of paranoia that were developed prior to and thus independently of the pandemic. As such, these studies have assessed instances of *general* paranoia during the pandemic (i.e. people have been talking about me behind my back; others want to hurt me), rather than *pandemic-specific* paranoia; that is, paranoid cognitions that focus specifically on the threat posed by others to oneself because of the pandemic (i.e. others spreading rumours that I have COVID-19; others wanting to infect me with COVID-19). We propose that the specific threat of being infected is likely to be reflected in the content of paranoid concerns and that this is likely to be related to but nevertheless distinct from general paranoia. This is supported by a general population survey of catastrophic cognitions (Rosebrook et al., 2021) of which some reflected paranoid concerns (i.e. others deliberately trying to give me the virus) that were moderately associated with general paranoia. A measure of pandemic-specific paranoia would have many possible benefits. Firstly, if pandemic-specific paranoia exists, a measure specifically tailored to assess this would have the necessary precision to understand and predict behaviour during the pandemic. Secondly, such a measure would enable an examination of whether, how, and for whom, the context of a pandemic has given rise to pandemic-specific paranoia and how this relates to and differs from a general tendency towards paranoia. Thirdly, it could help us to understand how pandemic paranoia relates to indices of distress and pro-health behaviours and the ways in which it may be functional and/or problematic in the context of the pandemic. Fourth, it could be used to evaluate the impact of support during the pandemic, and finally, it may help to anticipate individuals who may find increased social contacts more difficult, as restrictions ease.

To this end, the current paper describes the development and psychometric evaluation of the Pandemic Paranoia Scale (PPS), designed to measure pandemic-specific paranoia in the general population. Existing research suggests that paranoia is hierarchically structured in the general population, whereby common experiences of interpersonal worry, vulnerability, suspicion and mistrust, which are less explicitly persecutory, form the building blocks for delusional belief formation (Bebbington et al., 2013; Freeman et al., 2005). As the severity of threat of harm increases, thoughts become rarer and increasingly characterised by fears of intentional persecution and conspiracies. Informed by the paranoia hierarchy model and the approach to measuring general population paranoia taken by existing measures [e.g. The Revised Green et al., Paranoid Thoughts Scale (R-GPTS); Freeman et al., 2019] the PPS was designed to assess pandemic paranoia from across the full spectrum of experience, ranging from milder suspiciousness and mistrust (e.g. ‘Other people cannot be trusted to keep our community safe from COVID-19’) to more severe ideas of intentional persecution and conspiracy (e.g. ‘I couldn't stop thinking about people wanting to infect me with COVID-19’, ‘The government is using the COVID-19 pandemic to control us’). The current paper aimed to (1) determine the factor structure and item loadings for the PPS in a first representative sample from the UK, (2) use confirmatory factor analysis (CFA) to examine the factor structure and item loadings in a pooled sample from the four remaining sites (USA, Australia, Germany and Hong Kong) including three language versions (English, German and Chinese), (3) examine measurement variance across cultures/languages, (4) examine convergence of factors on a higher order factor and (5) examine psychometric properties (validity and reliability).

## Methods

### Design

A cross-sectional design was employed. The full sample (i.e. UK, USA, Germany, Hong Kong and Australia) completed the PPS at baseline, alongside construct validity measures (detailed below). Ninety-eight participants from the UK sample recompleted measures 3 months later, enabling examination of test–retest reliability. The construct validity was tested by examining its convergence with trait paranoia (medium-large positive correlation predicted), COVID-anxiety (medium positive correlation predicted), conspiracy beliefs (medium positive correlation predicted) and perceived risk of infection (medium positive correlation predicted).

### Participants

Participants were recruited via Qualtrics using stratified quota sampling to ensure that each sample was representative of the respective general population based on sex, age and educational attainment. Data were collected between February and March 2021. Sample size was determined using Everitt's (1975) minimum ratio recommendation of 10:1 (participants:scale items) per site. A total of 2510 participants met quota and quality assurance conditions (see section ‘Procedure’). The distribution across sites was: UK (*n* = 512), USA (*n* = 535), Germany (*n* = 516), Hong Kong (*n* = 445) and Australia (*n* = 502).

### Measures

*Pandemic Paranoia Scale (PPS)*. An expert international panel was convened to develop the initial set of items. The initial pool of items was generated based on the existing measures of general population paranoia (e.g. R-GPTS; Freeman et al., 2019) and aimed to capture the full spectrum of paranoia, from milder concerns about suspicion and mistrust in the context of COVID-19 to more severe fears of intentional persecution and conspiracies, whilst incorporating key dimensions (e.g. preoccupation, conviction, distress; Statham, Emerson, & Rowse, 2019). An initial pool of 28 items was developed [score range 0 (not at all) to 4 (totally)], which could be clustered into the following themes: the belief that others want to infect me with COVID-19 (e.g. *I was sure someone wanted to infect me with COVID-19*); belief that others are intentionally spreading the virus (e.g. *other people cannot be trusted to keep our community safe from COVID-19*); the belief that others are talking about you/watching you in the context of COVID (e.g. *people are watching me more closely due to COVID-19*); distrust in the government/conspiracy beliefs (e.g. *politicians are using COVID-19 to control us*). Items were translated into German and Chinese for these samples. Bilingual graduate/masters level students translated the English items into German/Chinese. Items were back-translated and checked by authors. Items requiring adaptation were discussed and resolved by consensus.

*Descriptive and socio-demographic variables*. Participants provided information on a range of sociodemographic variables. Those reported in this study included: age, gender, household income, employment status (in the last year), country of birth and current diagnosis of a mental health disorder (yes/no).

The remaining measures were used for construct validation.

*The Revised Green et al.*, *Paranoid Thoughts Scale* (Freeman et al., 2019) is an 18-item measure comprised of two subscales: ideas of reference (8 items) and paranoia and ideas of persecution (10 items). Items are rated on a 5-point scale of *0 – not at all* to *4 – totally* and exhibit reliability across the paranoia continuum. In the current sample alpha was 0.959.

*COVID-19 anxiety* (Shevlin et al., 2020) was assessed using a single-item visual analogue scale. Participants were asked: ‘How anxious are you about the coronavirus COVID-19 pandemic?’ rated from 0 to 100 with 100 indicating higher levels anxiety.

*Perceived risk of infection* (Shevlin et al., 2020). Perceived risk was assessed using two items, assessing perceived probability of contracting the virus (slider of 0–100%, in increments of 10, and across three times scales – in the next month, the next 3 months and next 6 months) and perceived consequences if infected (slider of 0–100, *not too bad*–*very bad*), in increments of 10, and across the same three time scales.

*Conspiracy Mentality Questionnaire* (Bruder, Haffke, Neave, Nouripanah, & Imhoff, 2013) is a five item, unidimensional measure of the general tendency to believe in conspiracy theories (i.e. ‘I think that *…*’). Items are rated on a Likert scale from *0% – certainly not* to *100% – certain*. In the current sample alpha was 0.905.

Validated German and Chinese language versions of all scales were used where available. Any remaining scales and items were translated from English by bilingual graduate/master's level students and back-translated and checked by authors.

### Procedure

Ethical approval was obtained from each of the five host sites. Potential participants were contacted by Qualtrics to take part. Consenting participants completed the questionnaires online via Qualtrics and were reimbursed for their time. Three months later, those opting into the follow-up received a link to the second set of measures. To prevent missing data, participants were required to respond to all questions on each page before progressing through the survey. To enhance the accuracy of the data, participants had to correctly respond to all of five attention checks that were distributed through the survey. Completion time was also monitored and those taking less than half of the median completion time were excluded. Participants with a geographical location that did not correspond with the stated location, and/or who did not consent to their data being used and/or dropped out without completing all measures were excluded at source by Qualtrics. Participants not fulfilling quota conditions were also excluded. Based on these criteria and conditions, *n* = 3555 participants were excluded at source by Qualtrics.

### Statistical analysis

Statistical analysis was carried out using R 3.6.1 and SPSS 26.0. First, preliminary statistics were computed to assess skew, kurtosis and to report on sample characteristics.

Second, to assess the factor structure of the PPS, the UK sample was used to conduct a non-graphical analysis of the Scree plot. The number of factors for subsequent analyses was based on eigenvalues (>mean eigenvalue), parallel analysis and optimal coordinates (Raîche, Walls, Magis, Ripoel, & Blais, 2013). Only those items loading over 0.600 on a factor were retained for further analyses. Next, exploratory factor analysis (EFA) was conducted in the UK sample, using principal axis factoring to assess data-driven clustering of questionnaire items. The aim, here, was to observe the number and nature of latent factors without imposing theory driven constraints. CFA was conducted using the combined sample of the remaining four sites (USA, Germany, Hong Kong and Australia) to examine whether the factor model determined using EFA in the UK sample showed sufficient fit in this independent set of data. Indicators for model fit were: the comparative fit index (CFI) and the Tucker–Lewis index (TLI; with CFI/TLI >0.95 indicating good fit and CFI/TLI >0.90 indicating a sufficient fit), the root mean squared error of approximation (RMSEA, with RMSEA <0.06 indicating a good fit and RMSEA <0.08 indicating a sufficient fit) and the standardised root mean square residual (SRMR, with SRMR <0.08 indicating a sufficient fit). To account for the often non-normal distribution of paranoid ideation, CFA was calculated with maximum likelihood estimation with robust standard errors and a Satorra–Bentler scaled test statistic.

Third, measurement invariance was assessed across all five samples by calculating and comparing four CFA. In a first CFA, sample was entered as a group variable to test whether the proposed factor structure fitted equally to all five samples (configural invariance). In the second CFA, factor loadings were fixed to be equal across samples (weak invariance). In the third CFA, factor loadings and intercepts were fixed (strong or scalar invariance, necessary to compare latent trait values between samples). In the fourth and final steps, loadings, intercepts and residuals were fixed (strict invariance, necessary to directly compare sum-scores from different samples). Invariance was evaluated based on the established thresholds (Putnick & Bornstein, 2016) for CFI (ΔCFI >−0.01 for invariance), RMSEA (ΔCFI <0.015 for invariance) and SRMR (ΔCFI <0.015 for invariance), as well as the Akaike information criterion (AIC) and the Bayesian information criterion (BIC).

Fourth, internal consistency was assessed using Cronbach's alpha and composite reliability (*ω*) (Netemeyer, Bearden, & Sharma, 2003). Coefficients greater than 0.70 were considered acceptable.

Fifth, convergent validity was assessed (using data from all sites) by examining the covariation of the PPS scale with other related, but distinct, constructs. Good convergence was expected to occur between the PPS and (a) general tendency to experience paranoia (R-GPTS, moderate-strong correlation anticipated), (b) COVID-19 anxiety (moderate correlation anticipated), (c) perceived risk of infection (moderate correlation expected) and (d) conspiracy mentality (moderate correlation expected). Criterion validity was examined by testing whether those participants self-reporting a mental health diagnosis had elevated levels of PPS than those reporting no diagnosis.

Finally, test–retest reliability was examined in a subsample (*n* = 98, UK sample) using intraclass correlations (ICCs), specifically: two-way mixed effects, single measurement absolute agreement ICCs (Koo & Li, 2016), correlating baseline scores and those obtained 3-months later.

## Results

### Preliminary statistics

Two thousand five hundred and ten participants met quota and provided valid data. Sample characteristics are reported in Table 1. Across sites, all items were positively skewed (*M*_skew_ = 1.94; range: 0.38–3.02) and most items showed excess kurtosis (*M*_kurtosis_ = 3.51, range: −1.14 to 8.48). Characteristics of the test–retest UK subgroup were: *M*_age_ = 47.54, 55% female, highest education (1% primary, 15% GCSE, 32% A-level, 36% Undergraduate Degree, 13% Masters, 3% Ph.D.), average household income (13% <£18 500, 39% £18 500–36 999, 21% £37 000–55 999, 12% £56 000–74 999, 6% £75 000–92 999, 4% £93 000–111 999 and 4% >£112 000), employment (45% full time, 18% part time, 17% retired, 6% unemployed, 9% home keeper and 4% disabled) and 13% reporting a current mental health diagnosis.

**Table 1.** Sociodemographic details (age, gender, education, income, employment and mental health diagnosis) across samples

	UK (*n* = 512)	USA (*n* = 535)	AU (*n* = 502)	GE (*n* = 516)	HK (*n* = 445)	Total (*n* = 2510)
Age, *M* years (s.d.)	41.91 (14.87)	47.65 (17.05)	44.75 (17.55)	42.00 (13.79)	39.64 (13.57)	43.32 (15.73)
Gender (%)						
Male	47.1	46.4	48.2	49.2	43.1	46.9
Female	52.7	52.7	50.8	50.0	56.6	52.5
Genderqueer	0	0.2	0.2	0.6	0	0.2
TransMale/Female	0	0.4	0.2	0.2	0.2	0.1
Other	0	0.4	0.4	0	0	0.2
Education						
Primary	0.4%	5.2%	0.8%	0.4%	2.5%	1.9%
Secondary or equivalent	19.7%	0%	15.5%	59.7%	28.8%	24.5%
A-level or equivalent	38.3%	34.4%	49.2%	12.8%	18.2%	30.8%
Bachelor degree or equivalent	30.3%	46.7%	28.9%	11.4%	39.8%	31.3%
Master's degree or equivalent	9.4%	11.0%	4.6%	14.5%	10.1%	10.0%
PhD or equivalent	2.0%	2.6%	1.0%	1.2%	0.7%	1.5%
Income						
Under £18 500	15.6%	26.7%	22.9%	20.9%	8.5%	19.3%
£18 500–36 999	39.8%	25%	27.1%	28.3%	22.2%	28.6%
£37 000–55 999	23.6%	16.1%	13.3%	23.4%	28.8%	20.8%
£56 000–74 999	11.5%	10.1%	13.3%	14.7%	11.7%	12.3%
£75 000–92 999	4.7%	6.9%	12.4%	6.2%	13.9%	8.6%
£93 000–111 999	2.1%	7.5%	7.4%	3.3%	8.3%	5.7%
£112 000+	2.5%	7.7%	3.6%	3.1%	6.5%	4.7%
Employment						
Full time	50.4%	40.9%	41.8%	50.2%	74.4%	50.9%
Part time	20.7%	8.8%	13.9%	17.6%	9.7%	14.2%
Retired	10.4%	0%	16.9%	8.7%	3.6%	7.9%
Unemployed (looking)	4.9%	4.9%	7.4%	6.2%	1.6%	5.1%
Military	0%	0%	0%	0.2%	0%	0.4%
Unemployed (not looking)	2.0%	22.1%	2.8%	1.7%	0.7%	6.1%
Home keeper/carer	5.7%	9.2%	7.2%	4.5%	1.3%	5.7%
Disabled	1.6%	4.7%	6.0%	2.5%	0%	3.0%
Training/school	4.3%	8.2%	4.0%	8.3%	8.8%	6.7%
Mental health diagnosis (% years)	12.3%	22.4%	41.8%	20.0%	7.2%	21%

### Factor analysis

Prior to EFA, the item-correlations were checked for multicollinearity (*r* > 0.80) due to excessive similarity in item content. Out of the 378 item correlations, nine correlations (2.3%) were above the 0.80 threshold (see online Supplementary Table S1 for a detailed list). Of these nine item pairs, five showed no substantial item overlap. Item content for the remaining four items was deemed sufficiently different to warrant including all items in the analysis (e.g. items 11 and 12 shared the same scenario – being targeted to be infected. However, item 11 ‘I couldn't stop thinking about people wanting to infect me with COVID-19’ focused on preoccupation, whereas item 12 ‘I was distressed by being targeted by people who wanted me to catch COVID-19’ focused on distress associated with the paranoid belief; see online Supplementary Table S2 for a full list of item pairs and analysis of their content).

**Fig. 1.**
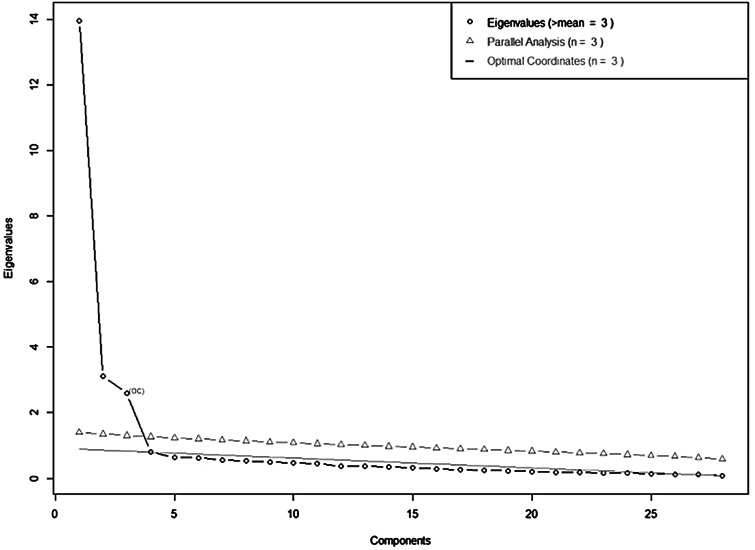
Eigenvalue-analysis, parallel analysis and optimal coordinates for EFA.

Eigenvalue-analysis, parallel analysis and optimal coordinates of the Scree plot converged on a three-factor solution (see Fig. 1; detailed results regarding the Scree plot analyses are found in online Supplementary Table S3). The results of a subsequent promax-rotated EFA are shown in Table 2. As can be seen, most items showed large loadings (>0.600) on one factor without any substantial cross loadings. The three factors accounted for 68.0% of the total item variance (F1: 0.392, F2: 0.170, F3: 0.110) and showed medium to large intercorrelation (*r*_F1,F2_ = 0.56, *r*_F1,F3_ = −0.51, *r*_F2,F3_ = −0.44). Three items showed no loading above 0.600 and were excluded from further analyses. The remaining 25 items also showed sufficiently high communality (>0.5 for all items, see Table 2). Thus, the resulting first factor *Persecutory Threat* comprised of 15 items focusing on beliefs about intentional actions aimed at harming the respondent. The second factor, *Paranoid Conspiracy*, included six items that captured beliefs that COVID-19 is a means of people in power controlling the general population. Finally, the factor *Interpersonal Mistrust* (four items) captured the lack of trust that other people will behave responsibly during the pandemic. The subsequent CFA on the four remaining samples (combined) showed sufficient model fit of the three-factor solution for the 25-item PPS across all fit indices (χ^2^(272) = 2880, *p* < 0.001, CFI = 0.949, TLI = 0.943, RMSEA = 0.041, SRMR = 0.049, BIC = 97 978), with high standardised loadings (range: 0.699–0.914). The three factorial model showed better fit than a one-dimensional model χ^2^(275) = 11 485, *p* < 0.001, CFI = 0.742, TLI = 0.719, RMSEA = 0.091, SRMR = 0.115, BIC = 106 561; χ^2^-difference test: χ^2^(3) = 8604, *p* < 0.001).

**Table 2.** Results of EFA (UK sample) and loadings from the CFA (USA, Germany, Australia and Hong Kong)

		EFA				CFA
No.	Item	Communality	1	2	3	loadings^a^
*Factor 1: Persecutory threat*						
2	People are deliberately trying to pass COVID-19 to me.	0.772	**0.948**	−0.108		0.866
5	People are spreading the rumour that I have COVID-19.	0.777	**0.935**			0.872
14	I can't stop worrying about other people spreading the rumour that I have COVID-19.	0.759	**0.926**			0.855
12	I was distressed by being targeted by people who wanted me to catch COVID-19.	0.795	**0.917**			0.906
11	I couldn't stop thinking about people wanting to infect me with COVID-19.	0.811	**0.908**			0.896
17	People have been hostile towards me on purpose because they think I have COVID-19.	0.798	**0.901**			0.900
21	Strangers and friends look at me critically because they think I have COVID-19.	0.785	**0.894**			0.891
24	People have tried to contaminate my face mask or other COVID-19 protective gear.	0.730	**0.892**			0.886
1	I was sure someone wanted to infect me with COVID-19.	0.659	**0.855**			0.868
10	I was convinced there was a conspiracy to get me to catch COVID-19.	0.707	**0.849**			0.850
23	Some people try to make it hard for me to get access to face coverings and other COVID-19 protective gear.	0.653	**0.817**			0.838
19	Other people are trying to harm me on purpose by not abiding to social distancing rules.	0.618	**0.709**		0.196	0.832
26	I feel threatened by people watching me more closely due to COVID-19.	0.613	**0.647**	0.150		0.815
4	I was certain that people did things to put me at risk of catching COVID-19.	0.537	**0.620**		0.208	0.775
18	People are watching me more closely due to COVID-19.	0.576	**0.602**		0.192	0.751
9	I feel threatened by other people wearing face masks.	0.528	0.580	0.220		
*Factor 2: Paranoid conspiracy*						
6	The government is using the COVID-19 pandemic to control us.	0.793		**0.947**		0.891
13	COVID-19 is a conspiracy by powerful people.	0.598		**0.916**		0.844
8	COVID-19 is a conspiracy to make us all feel threatened.	0.768		**0.890**	−0.119	0.867
3	The government is lying to us about COVID-19.	0.612	−0.108	**0.839**		0.832
7	The government is deciding things about COVID-19 behind our backs.	0.620	−0.181	**0.803**	0.156	0.812
22	Social distancing is a way to keep people under control by the government.	0.616		**0.763**		0.772
*Factor 3: Interpersonal mistrust*						
16	I can't trust others to stick to the social distancing rules.	0.609	−0.207	−0.112	**0.896**	0.738
27	Other people cannot be trusted to keep our community safe from COVID-19.	0.598			**0.817**	0.777
25	I can't stop worrying about other people failing to stick to the rules.	0.596			**0.795**	0.755
15	I need to be on my guard against others to protect myself from getting COVID-19.	0.542			**0.761**	0.700
20	I am distressed by people giving wrong information about COVID-19.	0.474		0.122	0.595	
28	I am angry that some people are trying to withhold important information about COVID-19 from me.	0.487	0.196	0.385	0.270	
	Sum of squared loadings		10.969	4.748	3.314	
	Proportion of variance explained		0.392	0.170	0.118	
	Cumulative variance		0.392	0.561	0.680	
Factor correlations (EFA/CFA)						
	Factor 2: Paranoid conspiracy		EFA: 0.556 CFA: 0.564			
	Factor 3: Interpersonal mistrust		EFA: −0.506 CFA: 0.541	EFA: −0.435 CFA: 0.382		

*Note*: Only loadings >0.100 are shown. Loadings printed in bold denote items included in the final version of the PPS.

a For a more precise estimation, the presented CFA loadings were estimated based on the full sample (UK, USA, Australia, Germany and Hong Kong). Factor correlations show the standardised linear correlations between the three factors in the EFA model (based on UK sample) and the CFA (based on combined USA, Australian, Germany and Hong Kong samples).

### Measurement invariance

The results of the measurement invariance analyses are summarised in Table 3. As can be seen, the configural invariance model showed sufficient fit, indicating an equal factor structure across different samples and language versions. Differences in the fit-indices remained in an acceptable area and the BIC was comparatively low for weak invariance. For the strong invariance and the strict invariance models, however, ΔCFI exceeded threshold values and AIC and BIC were larger compared to the configural invariance model. Thus, the data suggested weak invariance. Based on this, we continued to use the PPS factor scores for the criterion validation (see Table 1, right column for the item loadings from the full sample CFA).

**Table 3.** Results from the measurement invariance analyses

Fit index	Configural invariance model	Weak invariance model	Strong/scalar invariance model	Strict invariance model
CFI	0.924	0.921	0.901	0.856
ΔCFI		−0.003	**−0.020**	**−0.045**
TLI	0.916	0.919	0.904	0.868
RMSEA	0.044	0.043	0.047	0.055
ΔRMSEA		−0.001	0.004	0.008
SRMR	0.061	0.069	0.072	0.076
ΔSRMR		0.008	0.003	0.004
AIC	119 292	119 491	120 041	122 106
BIC	121 565	121 251	121 289	122 771
χ^2^	7118	7493	8219	10 484
df	1360	1448	1536	1636
χ^2^ diff		129.43	2141.26	856.81
df diff		88	88	100
*p* diff		<0.001	<0.001	<0.001

*Note*: ΔCFI/RMSEA/SRMR in bold indicate a difference in the respective fit index that exceeds the nested model-criteria for invariance.

### General pandemic paranoia factor

**Fig. 2.**
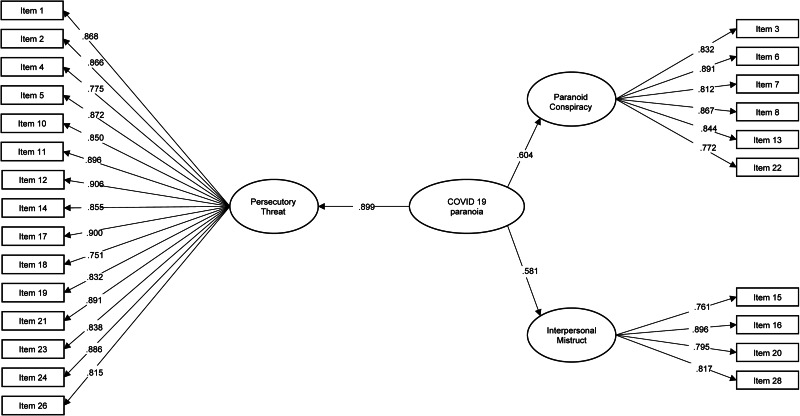
PPS factor structure.

To test whether the three PPS factors can be combined into one global indicator of pandemic paranoia, we repeated the CFA with an additional second-order factor. As can be seen in Fig. 2, all three factors showed large loadings on the second-order factor (Persecutory Threat: 0.899, Paranoid Conspiracy: 0.604 and Interpersonal Mistrust: 0.581).

Since three first-order factors lead to a just-identified second-order factor model, we imposed further constraint to the second-order factor model in the form of equal loadings of all first-order factors on the second-order factor. The model showed a sufficient fit in the four site sample (χ^2^(274) = 2898, *p* < 0.001, CFI = 0.948, TLI = 0.943, RMSEA = 0.041, SRMR = 0.058). The pattern of results for the measurement invariance analysis using the full five-site sample remained unchanged, with the configural invariance model (CFI = 0.922, TLI = 0.915, RMSEA = 0.044, SRMR = 0.070, AIC = 119 292, BIC = 121 565) and the metric invariance model (CFI = 0.920, TLI = 0.918, RMSEA = 0.043, SRMR = 0.076, AIC = 119 519, BIC = 121 220) showing the sufficient fit, but no scalar invariance as indicated by a change in CFI above 0.01 (CFI = 0.901, TLI = 0.903, RMSEA = 0.047, SRMR = 0.078, AIC = 120 077, BIC = 121 289).

### Internal consistency

Internal consistencies were good to excellent for Persecutory Threat (*α* = 0.97, *ω* = 0.98), Paranoid Conspiracy (*α* = 0.93, *ω* = 0.93) and Interpersonal Mistrust (*α* = 0.83, *ω* = 0.83) as well as the PPS Global score (*α* = 0.90, *ω* = 0.74).

### Convergent validity

The results of the convergent validation analyses are summarised in Table 4. As can be seen, all three PPS factors showed the expected moderate to strong associations with a general tendency to experience paranoia (i.e. paranoid ideation as well as ideas of reference). Interpersonal Mistrust showed the expected moderate association with COVID-19 anxiety and a small to moderate association between the perceived risk and severity of an infection, whereas Persecutory Threat showed a moderate association with the perceived personal risk of infection, but consistently small correlations with COVID-19 anxiety and perceived infection severity. Paranoid Conspiracy showed no substantial correlation with COVID-19 anxiety or the perceived risk variables. There was, however, a strong correlation between this factor and general conspiracy mentality, whereas the other factors showed only small associations with this criterion. Interestingly, R-GPTS scores were more highly correlated with the PPS Global scores than factor scores.

**Table 4.** Correlation between PPS factor scores and global scores and convergent validity criteria

	Persecutory threat		Paranoid conspiracy		Interpersonal mistrust		Pandemic Paranoia Global score	
	*r*	*p*	*r*	*p*	*r*	*p*	*r*	*p*
R-GPTS paranoid ideation	0.654	<0.001	0.458	<0.001	0.427	<0.001	0.678	<0.001
R-GPTS ideas of reference	0.574	<0.001	0.430	<0.001	0.424	<0.001	0.609	<0.001
COVID-anxiety	0.227	<0.001	−0.012	0.535	0.384	<0.001	0.223	<0.001
Perceived risk of infection								
Personal risk, 1 month	0.313	<0.001	0.064	0.001	0.299	<0.001	0.294	<0.001
Personal risk, 3 months	0.317	<0.001	0.070	0.001	0.314	<0.001	0.302	<0.001
Personal risk, 6 months	0.356	<0.001	0.115	<0.001	0.325	<0.001	0.344	<0.001
Infection severity, 1 month	0.134	<0.001	−0.043	0.033	0.271	<0.001	0.128	<0.001
Infection severity, 3 months	0.175	<0.001	−0.015	0.438	0.288	<0.001	0.169	<0.001
Infection severity; 6 months	0.183	<0.001	0.000	0.999	0.282	<0.001	0.178	<0.001
General conspiracy mentality	0.171	<0.001	0.538	<0.001	0.247	<0.001	0.317	<0.001

### Criterion validity

As a provisional examination of criterion validity, we compared scores across global and factor scores for those reporting a current mental health diagnosis (*n* = 528) *v.* not (*n* = 1982). Since there was no scalar invariance, we calculated the effect sizes per country and integrated them using a fixed effects model in order to achieve an unbiased estimate of the general effect size (based on Cohen's *d*; for the descriptive values and effect sizes per country, see online Supplementary Table S4). Across sites, PPS global sum scores were significantly higher in people self-reporting a current mental illness diagnosis (*d* = 0.173, *Z* = 3.38, *p* < 0.001, corresponding significant differences in four sites), as were Paranoid Conspiracy sum scores (*d* = 0.151, *Z* = 2.77, *p* = 0.006, corresponding significant differences in two sites) and Interpersonal Mistrust sum scores (*d* = 0.314, *Z* = 6.10, *p* < 0.001, corresponding significant differences in four sites). For Paranoid Threat, a minimal, non-significant average difference (*d* = 0.061, *Z* = 1.19, *p* = 0.234) emerged, but a pattern of significantly higher scores in people with a mental illness diagnosis was found in three of the five sites (see online Supplementary Table S4). Notably, the Australian sample showed some anomalous effects in that Paranoid Threat and Global scores were significantly higher in those *not* reporting an MH condition.

### Test–retest reliability

In the UK subgroup (*n* = 98), the relationship between baseline PPS scores and 3-month scores were: Persecutory Threat [ICC = 0.75; 95% confidence interval (CI) 0.65–0.83], Paranoid Conspiracy (ICC = 0.64; 95% CI 0.50–0.75), Interpersonal Mistrust (ICC = 0.57; 95% CI 0.41–0.69) and PPS Global scores (ICC = 0.72; 95% CI 0.60–0.80).

## Discussion

This paper reports on the development and validation of the PPS, a measure designed to assess paranoid thinking, from across the full spectrum of experience, and focusing specifically on the threat posed by others to oneself in the context of the pandemic. Factor analyses supported three highly related but distinguishable factors: *Persecutory Threat*, which converged most strongly with the R-GPTS, captured beliefs that others will intentionally cause one physical or psychological harm via the pandemic (e.g. deliberately infecting me, preventing me from protecting myself, spreading rumours I am infected, being hostile/critical towards me because they believe I am infected); *Paranoid Conspiracy*, which converged most strongly with the conspiracy mentality questionnaire, captured the belief that COVID-19 is a conspiracy generated by those in power (e.g. government, politicians) to threaten and/or control people and *Interpersonal Mistrust*, which converged most strongly with COVID-19 anxiety and perceived severity of illness if infected, captured concerns about not being able to trust others to follow government guidance and keep their communities safe. Internal consistency was good to excellent across global and factor scores. Criterion validity suggested the elevated levels of PPS global scores, interpersonal mistrust and paranoid conspiracy for participants who self-reported a current mental health diagnosis as compared to the those who did not. However, differences were not significant for persecutory threat. Anomalous findings arising for the Australian sample require further examination, including more fine-grained differentiation between mental disorders (i.e. psychotic *v.* non-psychotic disorders) in future studies.

Whilst the factors showed differential associations with other constructs, they nonetheless demonstrated large factor loadings onto a second-order factor model. Furthermore, the PPS global score was more strongly associated with persecutory beliefs than any individual factor. These data suggested that the factors assess distinguishable aspects of the overarching pandemic paranoia construct. The PPS global score may therefore most accurately capture the overall construct of pandemic paranoia, whereas the three first-order factors tap into interrelated facets of pandemic paranoia that crucially differ in the associated affective responses (e.g. the presence of fear).

Although many factors are likely to affect the experience of the pandemic across the sites here investigated (e.g. government, vaccine availability, health care systems, etc.), the measurement invariance data suggested a consistent factor structure across cultures and languages (English, German and Chinese). Based on the overall pattern of fit indices, however, using simple sum or factor scores to compare the absolute values across cultures/languages may be at risk for bias. The change in one answer category has the same meaning across sites, however the starting value for item ratings differed across sites (i.e. participants in one site tending to be more liberal for endorsing paranoid thoughts than participants from another site). In order to compare samples across countries/languages, we recommend – if possible – to calculate latent trait scores in a weak-invariance model as a starting point for cross-cultural analyses.

The findings should be considered in light of several limitations. Firstly, item generation was deductive rather than inductive; that is, PPS items were based on existing knowledge and measurement of paranoia in the general population, rather than through consultation with people experiencing paranoia during the pandemic. As such, aspects of pandemic-specific paranoia may have been missed and/or the existing items may have not optimally captured this construct. Inductive methods, such as quantitative methods, are well suited to generating new questionnaires; however, the time-sensitive nature of the pandemic prevented this. Relatedly, self-report measures of paranoia are unavoidably affected by measurement error. Triangulation methods, such as by assessing the convergence of PPS scores with, for example, clinician-based assessments, behavioural observations and/or information obtained from close others would be beneficial. Furthermore, consistent with several other paranoia scales, the PPS did not include reverse-coded items. Amongst other benefits, reverse coding helps to correct for acquiescence bias (Weijters & Baumgartner, 2012). Correlating the PPS with other non-reverse coded questionnaires is therefore likely to include some degree of acquiescence bias. Secondly, whilst we purposefully sampled a representative sample from each region, there are groups that are nonetheless under-represented, such as those without access to a computer and those with low literacy skills. The sample was also limited to those who were aware of and signed up to Qualtrics, which may introduce unknown bias. Finally, in keeping with general paranoia measures such as the R-GPTS and the Paranoia Checklist, the PPS aimed to capture paranoid beliefs across the full spectrum of experience, thus including items that are less explicitly persecutory (e.g. items from Interpersonal Mistrust subscale). Consistent with the paranoia hierarchy model, our findings suggest that these milder beliefs are part of a broader paranoia spectrum, but future research understanding the link between milder and more sever beliefs is required.

Notwithstanding these limitations, the current findings have several implications. Our data suggest that within the umbrella term of pandemic paranoia, there are three clusters of concerns, each of which may have different implications. The content of the items measuring persecutory threat concerns appear to align most closely with paranoid beliefs seen in clinical groups. Future research would benefit from examining this further, such as by testing whether threat concerns exhibit characteristics that are reminiscent of clinical paranoia (e.g. distressing, preoccupying, persistent and adversely impacting functioning) and/or share common correlates with clinical paranoia (e.g. negative core beliefs, depression, anxiety, stress, victimisation, minority status, etc.). This factor could prove to be clinically useful, such as by helping to identify those with elevated and/or persistent threat concerns. Furthermore, if such threat concerns are reminiscent of clinical paranoia, it is possible that they could be effectively managed using evidence-based treatments for paranoia (e.g. Ellett et al., 2020; Freeman et al., 2015). The second factor, which measured paranoid conspiracy concerns, may yield useful information for considering trust-building measures by governing authorities such as adopting transparency in communication with the general public and in decision-making processes. Our final factor, interpersonal mistrust, seems to relate to the trust that people have within their local communities for others to responsibly follow guidelines. This is likely to be more or less prevalent depending on how well governing authorities have the pandemic under control (e.g. current infection rates and vaccination rates) as well as the extent to which communities have united *v.* fractured in the context of the pandemic. It could be interesting to understand community-based factors that increase/decrease concerns about interpersonal mistrust, whether these concerns persist as the threat diminishes, and interventions that may help to reduce such concerns. Having established these factors, future research investigating the prevalence (Ellett et al., under review) and structure of pandemic paranoia is warranted.

In summary, preliminary examination of the psychometric properties of the PPS, across five international sites and three languages, using representative samples in terms of sex, age and educational attainment support the factorial stability, construct validity and internal reliability of the subscales. The PPS offers an exciting and unique opportunity to now examine pandemic paranoia more fully.

## Supplementary material

For supplementary material accompanying this paper visit https://doi.org/10.1017/S0033291721004633. click here to view supplementary material
