# Supplementary material for: The Pandemic Paranoia Scale (PPS): factor structure and measurement invariance across languages
Source: Psychol Med. 2021 Dec 9;53(6):2652–61. doi: 10.1017/S0033291721004633 (PMC8712962; doi:10.1017/S0033291721004633)
Supplement: Supplementary file 1 [file S0033291721004633sup001.docx]

Supplement Table 1-A.

Item correlations for EFA sample (UK-sample) item 1-14 with correlations >0.80 highlighted in bold

| Item | Item No. | 1 | 2 | 3 | 4 | 5 | 6 | 7 | 8 | 9 | 10 | 11 | 12 | 13 | 14 |
| --- | --- | --- | --- | --- | --- | --- | --- | --- | --- | --- | --- | --- | --- | --- | --- |
| I was sure someone wanted to infect me with COVID-19. | 1 |  |  |  |  |  |  |  |  |  |  |  |  |  |  |
| People are deliberately trying to pass COVID-19 to me. | 2 | **0.85** |  |  |  |  |  |  |  |  |  |  |  |  |  |
| The government is lying to us about COVID-19. | 3 | 0.31 | 0.29 |  |  |  |  |  |  |  |  |  |  |  |  |
| I was certain that people did things to put me at risk of catching COVID-19. | 4 | 0.65 | 0.66 | 0.32 |  |  |  |  |  |  |  |  |  |  |  |
| People are spreading the rumour that I have COVID-19. | 5 | 0.79 | 0.77 | 0.30 | 0.62 |  |  |  |  |  |  |  |  |  |  |
| The government is using the COVID-19 pandemic to control us. | 6 | 0.31 | 0.30 | 0.74 | 0.36 | 0.36 |  |  |  |  |  |  |  |  |  |
| The government is deciding things about COVID-19 behind our backs. | 7 | 0.24 | 0.23 | 0.74 | 0.26 | 0.29 | 0.72 |  |  |  |  |  |  |  |  |
| COVID-19 is a conspiracy to make us all feel threatened. | 8 | 0.35 | 0.37 | 0.65 | 0.36 | 0.41 | 0.75 | 0.61 |  |  |  |  |  |  |  |
| I feel threatened by other people wearing face masks. | 9 | 0.58 | 0.60 | 0.35 | 0.46 | 0.62 | 0.44 | 0.37 | 0.47 |  |  |  |  |  |  |
| I was convinced there was a conspiracy to get me to catch COVID-19. | 10 | 0.73 | 0.74 | 0.31 | 0.57 | 0.79 | 0.38 | 0.30 | 0.43 | 0.64 |  |  |  |  |  |
| I couldn’t stop thinking about people wanting to infect me with COVID-19. | 11 | 0.72 | 0.76 | 0.34 | 0.63 | 0.78 | 0.41 | 0.32 | 0.42 | 0.65 | 0.79 |  |  |  |  |
| I was distressed by being targeted by people who wanted me to catch COVID-19. | 12 | 0.69 | 0.80 | 0.32 | 0.64 | 0.74 | 0.37 | 0.29 | 0.42 | 0.63 | 0.74 | **0.82** |  |  |  |
| COVID-19 is a conspiracy by powerful people. | 13 | 0.37 | 0.38 | 0.66 | 0.33 | 0.43 | 0.78 | 0.65 | **0.85** | 0.49 | 0.45 | 0.46 | 0.41 |  |  |
| I can’t stop worrying about other people spreading the rumour that I have COVID-19 | 14 | 0.68 | **0.81** | 0.28 | 0.57 | 0.80 | 0.33 | 0.26 | 0.43 | 0.56 | 0.67 | 0.75 | **0.81** | 0.43 |  |
| I need to be on my guard against others to protect myself from getting COVID-19. | 15 | 0.26 | 0.27 | 0.17 | 0.36 | 0.24 | 0.18 | 0.26 | 0.19 | 0.30 | 0.23 | 0.29 | 0.28 | 0.22 | 0.26 |
| I can’t trust others to stick to the social distancing rules. | 16 | 0.16 | 0.14 | 0.13 | 0.27 | 0.11 | 0.08 | 0.27 | 0.06 | 0.10 | 0.12 | 0.15 | 0.16 | 0.07 | 0.13 |
| People have been hostile towards me on purpose because they think I have COVID-19. | 17 | 0.68 | 0.73 | 0.28 | 0.59 | 0.78 | 0.41 | 0.30 | 0.45 | 0.59 | 0.77 | **0.83** | 0.78 | 0.48 | 0.76 |
| People are watching me more closely due to COVID-19. | 18 | 0.57 | 0.62 | 0.34 | 0.55 | 0.61 | 0.37 | 0.34 | 0.42 | 0.50 | 0.64 | 0.65 | 0.68 | 0.41 | 0.63 |
| Other people are trying to harm me on purpose by not abiding to social distancing rules. | 19 | 0.65 | 0.72 | 0.28 | 0.67 | 0.64 | 0.31 | 0.26 | 0.35 | 0.50 | 0.58 | 0.65 | 0.70 | 0.37 | 0.69 |
| I am distressed by people giving wrong information about COVID-19. | 20 | 0.34 | 0.34 | 0.29 | 0.38 | 0.33 | 0.30 | 0.40 | 0.29 | 0.40 | 0.32 | 0.36 | 0.34 | 0.35 | 0.31 |
| Strangers and friends look at me critically because they think I have COVID-19. | 21 | 0.66 | 0.73 | 0.31 | 0.63 | 0.77 | 0.39 | 0.33 | 0.44 | 0.60 | 0.72 | **0.81** | 0.79 | 0.45 | 0.77 |
| Social distancing is a way to keep people under control by the government. | 22 | 0.34 | 0.34 | 0.54 | 0.31 | 0.37 | 0.71 | 0.53 | 0.69 | 0.48 | 0.41 | 0.42 | 0.40 | 0.73 | 0.36 |
| Some people try to make it hard for me to get access to face coverings and other COVID-19 protective gear. | 23 | 0.58 | 0.64 | 0.26 | 0.52 | 0.65 | 0.37 | 0.29 | 0.39 | 0.62 | 0.62 | 0.73 | 0.76 | 0.40 | 0.71 |
| People have tried to contaminate my face mask or other COVID-19 protective gear. | 24 | 0.64 | 0.71 | 0.23 | 0.57 | 0.78 | 0.38 | 0.26 | 0.41 | 0.58 | 0.70 | 0.78 | 0.71 | 0.44 | 0.74 |
| I can’t stop worrying about other people failing to stick to the rules. | 25 | 0.29 | 0.31 | 0.15 | 0.39 | 0.28 | 0.18 | 0.26 | 0.19 | 0.28 | 0.27 | 0.32 | 0.33 | 0.21 | 0.29 |
| I feel threatened by people watching me more closely due to COVID-19. | 26 | 0.57 | 0.65 | 0.34 | 0.54 | 0.66 | 0.41 | 0.34 | 0.51 | 0.66 | 0.62 | 0.68 | 0.71 | 0.48 | 0.65 |
| Other people cannot be trusted to keep our community safe from COVID-19. | 27 | 0.23 | 0.25 | 0.18 | 0.33 | 0.25 | 0.19 | 0.25 | 0.19 | 0.25 | 0.26 | 0.29 | 0.25 | 0.23 | 0.23 |
| I am angry that some people are trying to withhold important information about COVID-19 from me. | 28 | 0.36 | 0.40 | 0.45 | 0.44 | 0.40 | 0.50 | 0.47 | 0.51 | 0.44 | 0.49 | 0.51 | 0.50 | 0.53 | 0.41 |

Supplement Table 1-B.

Item correlations for EFA sample (UK-sample) item 15-27

| Item | Item No. | 15 | 16 | 17 | 18 | 19 | 20 | 21 | 22 | 23 | 24 | 25 | 26 | 27 |
| --- | --- | --- | --- | --- | --- | --- | --- | --- | --- | --- | --- | --- | --- | --- |
| I can’t trust others to stick to the social distancing rules. | 16 | 0.60 |  |  |  |  |  |  |  |  |  |  |  |  |
| People have been hostile towards me on purpose because they think I have COVID-19. | 17 | 0.29 | 0.13 |  |  |  |  |  |  |  |  |  |  |  |
| People are watching me more closely due to COVID-19. | 18 | 0.35 | 0.25 | 0.66 |  |  |  |  |  |  |  |  |  |  |
| Other people are trying to harm me on purpose by not abiding to social distancing rules. | 19 | 0.36 | 0.25 | 0.67 | 0.64 |  |  |  |  |  |  |  |  |  |
| I am distressed by people giving wrong information about COVID-19. | 20 | 0.50 | 0.44 | 0.36 | 0.45 | 0.44 |  |  |  |  |  |  |  |  |
| Strangers and friends look at me critically because they think I have COVID-19. | 21 | 0.30 | 0.16 | **0.83** | 0.66 | 0.68 | 0.38 |  |  |  |  |  |  |  |
| Social distancing is a way to keep people under control by the government. | 22 | 0.25 | 0.14 | 0.43 | 0.40 | 0.31 | 0.30 | 0.40 |  |  |  |  |  |  |
| Some people try to make it hard for me to get access to face coverings and other COVID-19 protective gear. | 23 | 0.30 | 0.11 | 0.74 | 0.59 | 0.59 | 0.30 | 0.78 | 0.40 |  |  |  |  |  |
| People have tried to contaminate my face mask or other COVID-19 protective gear. | 24 | 0.28 | 0.09 | **0.81** | 0.55 | 0.61 | 0.29 | 0.80 | 0.41 | 0.77 |  |  |  |  |
| I can’t stop worrying about other people failing to stick to the rules. | 25 | 0.54 | 0.54 | 0.28 | 0.39 | 0.41 | 0.54 | 0.30 | 0.21 | 0.30 | 0.30 |  |  |  |
| I feel threatened by people watching me more closely due to COVID-19. | 26 | 0.31 | 0.18 | 0.69 | 0.61 | 0.58 | 0.41 | 0.66 | 0.45 | 0.65 | 0.63 | 0.35 |  |  |
| Other people cannot be trusted to keep our community safe from COVID-19. | 27 | 0.55 | 0.62 | 0.30 | 0.34 | 0.33 | 0.47 | 0.28 | 0.25 | 0.26 | 0.27 | 0.65 | 0.32 |  |
| I am angry that some people are trying to withhold important information about COVID-19 from me. | 28 | 0.37 | 0.27 | 0.52 | 0.53 | 0.45 | 0.45 | 0.47 | 0.50 | 0.46 | 0.45 | 0.40 | 0.52 | 0.37 |

Supplement Table 2

Content comparison of item pairs with high correlation (>0.80)

| Item 1 | Item 2 | Notable overlap in item content | Notable differences in item content |
| --- | --- | --- | --- |
| I was sure someone wanted to infect me with COVID-19. | People are deliberately trying to pass COVID-19 to me. | similar scenario (deliberate harm) | Different perpetrator (single person vs group of infected) |
| People are deliberately trying to pass COVID-19 to ,me | I can’t stop worrying about other people spreading the rumor that I have COVID-19 | None | different scenarios (deliberate harm vs. spreading rumors), different focus (belief in paranoid idea vs. cognitive-emotional response (worrying)) |
| I couldn’t stop thinking about people wanting to infect me with COVID-19. | I was distressed by being targeted by people who wanted me to catch COVID-19. | similar scenario (deliberate harm) | different focus (preoccupation with paranoid belief vs distress due to paranoid belief) |
| I couldn’t stop thinking about people wanting to infect me with COVID-19. | People have been hostile towards me on purpose because they think I have COVID-19. | None | different scenarios (deliberate harm vs. paranoid social evaluative concerns) |
| I couldn’t stop thinking about people wanting to infect me with COVID-19. | Strangers and friends look at me critically because they think I have COVID-19. | None | different scenarios (deliberate harm vs. paranoid social evaluative concerns) |
| COVID-19 is a conspiracy to make us all feel threatened. | COVID-19 is a conspiracy by powerful people. | similar scenario (large scale conspiracy) | Focus on motivator for conspiracy vs participants in conspiracy) |
| I was distressed by being targeted by people who wanted me to catch COVID-19. | I can’t stop worrying about other people spreading the rumor that I have COVID-19. | None | different scenarios (deliberate harm vs. paranoid social evaluative concerns) |
| People have been hostile towards me on purpose because they think I have COVID-19. | Strangers and friends look at me critically because they think I have COVID-19. | similar scenario (paranoid social evaluative concerns) | different grading (mild negative response vs. open hostility) |
| People have been hostile towards me on purpose because they think I have COVID-19. | People have tried to contaminate my face mask or other COVID-19 protective gear. | None | different scenarios (deliberate harm vs. paranoid social evaluative concerns) |

Supplement Table 3.

Eigenvalues of all components from the Prinicipal Component Analysis and simulated eigenvalues from Parallel Analysis

| Component | Eigenvalue | Expl. Variance | Simulated Eigenvalues (Parallel Analysis) | Predicted eigenvalue (optimal coordinates) |
| --- | --- | --- | --- | --- |
| 1 | 13.954 | 0.498 | 1.400 | 3.226 |
| 2 | 3.109 | 0.111 | 1.346 | 2.699 |
| 3 | 2.599 | 0.093 | 1.302 | 0.833 |
| 4 | 0.803 | 0.029 | 1.263 | 0.668 |
| 5 | 0.643 | 0.023 | 1.229 | 0.648 |
| 6 | 0.623 | 0.022 | 1.196 | 0.584 |
| 7 | 0.561 | 0.020 | 1.165 | 0.548 |
| 8 | 0.525 | 0.019 | 1.135 | 0.522 |
| 9 | 0.500 | 0.018 | 1.107 | 0.484 |
| 10 | 0.462 | 0.017 | 1.079 | 0.480 |
| 11 | 0.458 | 0.016 | 1.053 | 0.391 |
| 12 | 0.372 | 0.013 | 1.026 | 0.385 |
| 13 | 0.365 | 0.013 | 1.001 | 0.365 |
| 14 | 0.345 | 0.012 | 0.974 | 0.338 |
| 15 | 0.319 | 0.011 | 0.948 | 0.305 |
| 16 | 0.287 | 0.010 | 0.925 | 0.272 |
| 17 | 0.256 | 0.009 | 0.899 | 0.266 |
| 18 | 0.249 | 0.009 | 0.874 | 0.243 |
| 19 | 0.227 | 0.008 | 0.850 | 0.219 |
| 20 | 0.203 | 0.007 | 0.826 | 0.201 |
| 21 | 0.186 | 0.007 | 0.800 | 0.195 |
| 22 | 0.178 | 0.006 | 0.774 | 0.180 |
| 23 | 0.163 | 0.006 | 0.750 | 0.172 |
| 24 | 0.153 | 0.005 | 0.722 | 0.160 |
| 25 | 0.139 | 0.005 | 0.694 | 0.151 |
| 26 | 0.126 | 0.005 | 0.664 | 0.156 |
| 27 | 0.116 | 0.004 | 0.629 | NA |
| 28 | 0.077 | 0.003 | 0.582 | NA |

Supplement Table 4

Comparison of PPS sum scores between people with and without a current mental illness diagnosis

|  | Site | UK | | USA | | Australia | | Germany | | Hong Kong | |
| --- | --- | --- | --- | --- | --- | --- | --- | --- | --- | --- | --- |
|  | Mental Illness | Yes | No | Yes | No | Yes | No | Yes | No | Yes | No |
| PPS score | Group Size | 63 | 449 | 120 | 415 | 210 | 292 | 103 | 413 | 32 | 413 |
| Paranoid Threat | M | 3.556 | 2.858 | 8.525 | 4.684 | 5.067 | 10.914 | 5.515 | 3.254 | 11.969 | 5.782 |
|  | SD | 8.811 | 8.167 | 15.916 | 11.288 | 12.281 | 15.14 | 10.781 | 7.584 | 14.273 | 10.162 |
|  | d | 0.085 | | **0.308** | | **-0.417** | | **0.272** | | **0.589** | |
|  | 95%-CI | -0.179; 0.348 | | **0.104; 0.512** | | **-0.596: -0.238** | | **0.055; 0.488** | | **0.227; 0.951** | |
| Paranoid Conspiracy | M | 5.667 | 4.221 | 7.417 | 5.116 | 5.967 | 7.041 | 5.738 | 4.63 | 9.969 | 7.005 |
|  | SD | 6.312 | 5.676 | 7.832 | 6.756 | 7.543 | 6.728 | 6.636 | 6.084 | 6.572 | 6.357 |
|  | d | 0.251 | | **0.328** | | -0.152 | | 0.179 | | **0.465** | |
|  | 95%-CI | -0.013; 0.515 | | **0.124; 0.532** | | -0.329; 0.026 | | -0.037; 0.395 | | **0.104; 0.826** | |
| Interpersonal Mistrust | M | 6.921 | 4.938 | 6.275 | 4.248 | 5.186 | 5.767 | 5.777 | 3.472 | 7.344 | 4.593 |
|  | SD | 4.382 | 4.288 | 5.026 | 4.42 | 4.563 | 4.223 | 4.653 | 3.745 | 4.132 | 3.596 |
|  | d | **0.461** | | **0.444** | | -0.133 | | **0.585** | | **0.757** | |
|  | 95%-CI | **0.196; 0.726** | | **0.239; 0.649** | | -0.311; 0.045 | | **0.366; 0.804** | | **0.394; 1.120** | |
| Global score | M | 16.143 | 12.016 | 22.217 | 14.048 | 16.219 | 23.723 | 17.029 | 11.356 | 29.281 | 17.38 |
|  | SD | 13.703 | 14.446 | 24.282 | 18.11 | 20.359 | 22.957 | 17.774 | 13.237 | 21.708 | 16.695 |
|  | d | **0.287** | | **0.416** | | **-0.343** | | **0.398** | | **0.696** | |
|  | 95%-CI | **0.023; 0.552** | | **0.211; 0.620** | | **-0.521; -0.164** | | **0.181; 0.615** | | **0.334; 1.059** | |

Note. 95%-CI= 95% Confidence interval of the effect size Cohen’s d. Significant differences between participants with and without current mental illness diagnosis are printed bold, with black bold differences indicating results in the expected direction (participants with mental illness diagnosis showing higher scores) and red bold differences indicating results in the opposite direction (healthy comparison participants showing higher scores)
